# Inertial Sensors and Pressure Pain Threshold to Evaluate People with Primary Adhesive Capsulitis: Comparison with Healthy Controls and Effects of a Physiotherapy Protocol

**DOI:** 10.3390/jfmk8040142

**Published:** 2023-10-06

**Authors:** Manuela Deodato, Miriam Martini, Alex Buoite Stella, Giulia Citroni, Miloš Ajčević, Agostino Accardo, Luigi Murena

**Affiliations:** 1 Department of Medicine, Surgery, and Health Sciences, School of Physiotherapy, University of Trieste, Via Pascoli 31, 34100 Trieste, Italy; mdeodato@units.it (M.D.); miriam.martini@live.it (M.M.); giulia.citroni@gmail.com (G.C.); lmurena@units.it (L.M.); 2 Department of Engineering and Architecture, University of Trieste, Via Alfonso Valerio 10, 34100 Trieste, Italy; majcevic@units.it (M.A.); accardo@units.it (A.A.); 3 Orthopedics and Traumatology Unit, Department of Medicine, Surgery, and Health Sciences, Cattinara Hospital—ASUGI, Strada di Fiume 447, 34149 Trieste, Italy

**Keywords:** adhesive capsulitis, scapular dyskinesia, range of motion, pressure pain threshold, kinematic

## Abstract

Inertial sensors (IMUs) have been recently widely used in exercise and rehabilitation science as they can provide reliable quantitative measures of range of motion (RoM). Moreover, the pressure pain threshold (PPT) evaluation provides an objective measure of pain sensation in different body areas. The aim of this study was to evaluate the efficacy of physiotherapy treatment in people with adhesive capsulitis in terms of RoM and pain improvement measured by IMUs and the PPT. A combined prospective cohort/cross-sectional study was conducted. Nineteen individuals with adhesive capsulitis (10/19 females, 54 ± 8 years) and nineteen healthy controls (10/19 females, 51 ± 6 years) were evaluated for active glenohumeral joint RoM and PPT on shoulder body areas. Then, individuals with adhesive capsulitis were invited to 20 sessions of a physiotherapy protocol, and the assessments were repeated within 1 week from the last session. The range of motion in the flexion (*p* = 0.001) and abduction (*p* < 0.001) of the shoulder increased significantly after the physiotherapy protocol. Similarly, the PPT was found to increase significantly in all the assessed shoulder body areas, leading to no significant differences compared to the healthy controls. IMU and PPT assessments could be used to evaluate the efficacy of physical therapy in people with adhesive capsulitis.

## 1. Introduction

Adhesive capsulitis represents the most common musculoskeletal condition of shoulder pain and dysfunction [1,2]. This condition is also called “frozen shoulder” due to its clinical and pathophysiological manifestations; indeed, adhesive capsulitis is characterized by a pathophysiological course from capsular inflammation to fibrosis that is characterized by a clinical progression from severe pain symptoms to an important reduction in the range of motion (RoM) [3,4,5,6]. Based on these clinical and pathophysiological manifestations, physiotherapy treatment can play an important role in addressing the issue [7].

Traditionally, the first approach to this syndrome is a conservative treatment that combines physiotherapy with intra-articular injections and oral medications [1,3,8,9,10,11]. Despite the numerous studies in the literature concerning the treatments, the results are still inconsistent and controversial. On one side, the treatment strategies of adhesive capsulitis are still so different from each other, and there are still no defined protocols. On the other side, the pathogenesis and natural history of adhesive capsulitis are still poorly understood and unclear [3,7,12,13,14]. Today, extensive research has shown the efficacy of physiotherapy in the decrease in pain, in the improvement in the range of motion, and in the functional status of patients affected by adhesive capsulitis [13]. The physiotherapy treatment includes different active and passive interventions: stretching exercises, extracorporeal shock wave therapy, laser therapy, ultrasound, cryotherapy, joint mobilization, muscle energy techniques, proprioceptive neuromuscular facilitation, continuous passive motion, strengthening of muscles, dynamic scapular recognition exercises, and manual muscle release techniques [15,16,17,18,19,20,21,22,23,24]. Generally, it is recommended to use a combination of these physiotherapy interventions despite a single intervention [13,21,23]. Nevertheless, the most effective interventions in adhesive capsulitis remain uncertain.

To improve the quality of data regarding the effectiveness of the physiotherapy treatment, it is important to identify the best evaluation setting to record the change in the symptomatology of people with adhesive capsulitis.

Therefore, RoM and pain represent the most important outcomes to consider in the treatment of adhesive capsulitis shoulder; in fact, many studies report these as the main outcomes [13]. It is, therefore, important to individualize the best tools to investigate and quantify the change in RoM and pain after the application of any interventions. Often, shoulder pain is measured by any kind of pain scale, but despite the good reliability coefficients and good internal reliability, these scales are strongly influenced by the single subject, guaranteeing only moderate accuracy and allowing the measurement of only self-reported pain [21,25]. Identifying the different tools to provide a measure of pain is often complicated due to their different dimensions: physical, sensory, behavioral, sociocultural, cognitive, affective, and spiritual [26]. Despite not being able to identify each of these dimensions, several studies have started to use the pressure pain threshold (PPT), assessed by using a pressure algometer, to provide an objective measure of peripheral pain [27,28,29]. This tool allows the evaluation of the subject’s tolerance to nociceptive stimuli, allowing the detection and quantification of the soreness of the investigated tissues by measuring the patient’s pain threshold to pressure stimuli (PPT) and pain sensitivity (PPS) with excellent reliability [30]. Nevertheless, a discrepancy between the PPT and subjective recordings of pain intensity might be present due to the elaboration of the pain modulation above the level of the spinal cord [31].

The range of motion assessment is usually measured with conventional goniometry due to its portability and low cost. Despite there being a trend for good reliability with goniometry measurement, a limitation of this tool is that it requires the clinician to use both hands, making stabilization of the extremity more difficult and thus increasing the risk of error in reading the instrument [32]. The use of IMMS (Inertial and Magnetic Measurement System) technology makes it possible to combine easy accessibility due to low cost and ease of transport and use while performing motion analysis with higher reliability, reducing the risk of error on the part of the operator performing the measurement. MTw sensors (Xsens Technologies, NL) allow for more complex and advanced data acquisition than just the RoM measured by a goniometer. In fact, these instruments are able to measure acceleration, angular velocity, and magnetic field intensity in the three orthogonal axes thanks to the presence of a 3D accelerometer, a 3D gyroscope, and a magnetometer, ensuring not only a quantitative assessment of movements but a qualitative one as well. In addition, such a system allows dynamic detection of scapulohumeral rhythm [33,34].

To date, no previous study has used a digital algometer and inertial sensors in combination to investigate the effect of a specific combined protocol of physiotherapy concerning the quality of movement and the pain threshold in patients with adhesive capsulitis. Therefore, the first aim of the present study is to evaluate the effect of a combined protocol of physiotherapy in individuals with adhesive capsulitis concerning kinematic and clinical parameters. The second aim is to compare the kinematic and clinical parameters in the shoulders of patients with adhesive capsulitis with respect to the shoulders of healthy controls before and after a combined protocol of physiotherapy.

## 2. Materials and Methods

### 2.1. Study Design

A combined prospective cohort/cross-sectional study was conducted over a period of 1 year, from December 2020 to December 2021, in a University Hospital setting. Nineteen individuals with a diagnosis of primary adhesive capsulitis were recruited from the Orthopedics and Traumatology Unit. Inclusion criteria were as follows: participants of both sexes, from 18 to 60 years, and with a diagnosis of adhesive capsulitis based on a physical examination to detect pain and RoM alterations that was supported by imaging to exclude some other possible conditions with similar clinical characteristics, such as osteoarthritis or chronic anterior or posterior dislocation [35]. Participants were excluded in case of pregnancy during the study; serious psychiatric pathologies; significant surgical procedures to the shoulder during the previous 12 months; contraindications to the rehabilitation treatment; serious pathologies such as traumas, tumors, or infections; and physical therapy or other conservative treatments in the previous 3 months. In particular, differential diagnosis excluded other conditions such as major trauma, rotator cuff tear, rotator cuff contusion, labral tear, bone contusion, subacromial bursitis, and cervical or peripheral neuropathy [35].

Nineteen healthy individuals with no previous history of shoulder traumas or other diseases and with similar demographics and anthropometrical characteristics were recruited and included in the study as a control group.

All the included participants were invited to a testing session performed at the Orthopedics and Traumatology unit to evaluate pain and kinematic parameters (t0). Then, the adhesive capsulitis group volunteered for 20 sessions of a physiotherapy protocol, including both manual therapy and active exercises, and the same assessments were repeated within one week from the end of the last treatment session (t1). All the assessments were performed at the same time of the day, in the afternoon, and asked the participants to avoid exercise or pain-modulating drugs in the previous 24 h.

The study was approved by the institutional review board (CEUR-2020-Os-246), and it was performed in accordance with the Declaration of Helsinki. The informed consent was obtained from all subjects, and the privacy rights of all subjects were protected.

### 2.2. Data Measurement

#### 2.2.1. ISEO Protocol

The RoM was assessed with inertial sensors according to the ISEO protocol [33,36]. Elevation, abduction, and scapulohumeral rhythm were measured both in the adhesive capsulitis group and in the healthy control group. ISEO protocol was selected for its reliability, validity, and repeatability (see Supplementary Materials). The ISEO protocol required MTw wireless sensor units (Xsens Technologies, Enschede, The Netherlands) for the acquisition of the kinematic signals and parameters. The orientation of the coordinate system of the MTw with respect to the earth-based coordinate system is provided by 3D gyroscopes, accelerometers, and magnetometers contained in each MTw sensor. The setting of the ISEO protocol procedure included a PC connected with 4 MTw wireless sensors applied to the skin of the subjects as follows: one on the thorax at the manubrium of the sternum; one above the scapulae, over the central third between the angulus acromialis and the trigonum spinae; one over the central third of the humerus; and one on the wrist. Before the data acquisition, sensor-to-segment calibration is necessary for the anatomical coordinate systems. The subjects are asked to maintain a static standing posture with the elbow flexed at 90°. After that, subjects were instructed to perform the flexion and the abduction of the arm correctly. Three consecutive assessments were taken for each movement, and then the average of the three evaluations was considered [33,36]. The adhesive capsulitis group participated in the ISEO protocol before (t0) and after each physiotherapy treatment (t1), while the healthy control group received the evaluation only once (t0).

#### 2.2.2. Pressure Pain Threshold

To investigate musculoskeletal pain sensitivity to mechanical stimuli, PPT was assessed with an algometer (Somedic Sales, Hörby, Sweden) on different body areas of the shoulder such as the sub-occipitalis, levator scapulae, subscapularis, and pectoralis minor. These four muscle groups were evaluated on the painful side of the body in the adhesive capsulitis group and the corresponding side in the healthy controls group; in particular, the suboccipital muscles were evaluated in their insertional component at the level of the occiput, the levator scapulae muscle in its distal portion at the level of the insertion of the superomedial angle of the scapula, the subscapularis muscle on its muscle belly, and the pectoralis minor muscle near its insertion on the coracoid process. The algometer was placed with the probe (circular 1 cm^2^) against the muscle belly, according to standard procedures, and pressure was increased at a rate of 30 kPa/s [27,28]. Participants were instructed to press a button as soon as they perceived a painful sensation on the tested body area, and the pressure value was automatically saved in the dedicated software. Before starting the muscle evaluation, the first trial was applied on the wrists of each subject to educate with the algometer assessment. Three measurements were performed on the shoulder with capsulitis and the corresponding shoulder in the healthy individuals, with 30 s of rest between each assessment [27,37]. The mean value was calculated and considered in the final analysis. The adhesive capsulitis group received the algometer assessment before (t0) and at the end of the physiotherapy treatment (t1), while the healthy control only once (t0).

#### 2.2.3. Physiotherapy Protocol

The physiotherapy protocol consisted of a combined treatment of manual therapy and active exercises [38]. It was scheduled as 20 one-hour individual sessions twice a week for three months. The manual therapy techniques started from the micro-mobilization of the areas biomechanically connected to the shoulder, such as the cervical and dorsal spine: central posterior–anterior mobilizations were used on the spinous processes from C2 to D12 segments [20,39,40]. Next, accessory shoulder joints were micro-mobilized (clavicula, acromioclavicular and sternoclavicular joints, scapula, and humeral head). Then, proprioceptive neuromuscular facilitation (PNF) techniques hold–relax and contract–relax (post-isometric relaxation) were applied to all movement directions of the shoulder (anterior flexion, abduction, external rotation, and internal rotation); the duration of contraction was from 5 to 10 s, and the duration of relaxation was from 10 to 20 s [7,41]. With regard to active exercise, a progression of active exercises was performed with a specific goal: to improve the scapulohumeral rhythm and the performance of the shoulder. Each exercise was performed under the supervision of a physiotherapist, and only later, once the exercises had been learned correctly, were exercises performed without further supervision. The protocol started with a proprioceptive exercise on the scapula to increase awareness of the scapular movements in the medial and lateral spaces, above and below, respectively [42]. Next, active exercises were presented progressively below 90°, at 90°, and over 90° of flexion and abduction of the shoulder to restore the correct scapulohumeral time of activation. In particular, circular, straight, and curved trajectories were used first without weights and progressively with weights and rubber bands. All manual therapy and active exercise sessions were performed by the same physical therapist.

### 2.3. Statistical Analysis

GraphPad InStat 3.06 was used, and the statistical significance level was ɑ 95% (0.05). t0 and t1 within treatments were compared with the Wilcoxon non-parametric test, while the differences between groups were calculated with the Mann–Whitney Test. Finally, the data were graphically processed with GraphPad Prism 8.4.1.

## 3. Results

A total of 38 subjects were enrolled: 19 patients with adhesive capsulitis and 19 healthy controls. The adhesive capsulitis group consisted of 10 women and 9 men with a mean age of 54 (SD 8); the healthy controls consisted of 10 women and 9 men with a mean age of 51 (SD 6). At baseline (t0), no statistical differences were found between the two groups in terms of age (*p* = 0.20), but statistical differences were found between the two groups in terms of abduction (*p* < 0.0001) and flexion (*p* < 0.0001) RoM, scapula (*p* < 0.001) and humerus (*p* < 0.01) timing of activation, as well as the PPT in the sub-occipitalis (*p* = 0.03), levator scapulae (*p* < 0.01), subscapularis (*p* = 0.02), and pectoralis minor (*p* = 0.001).

### 3.1. Range of Motion

**Figure 1:**
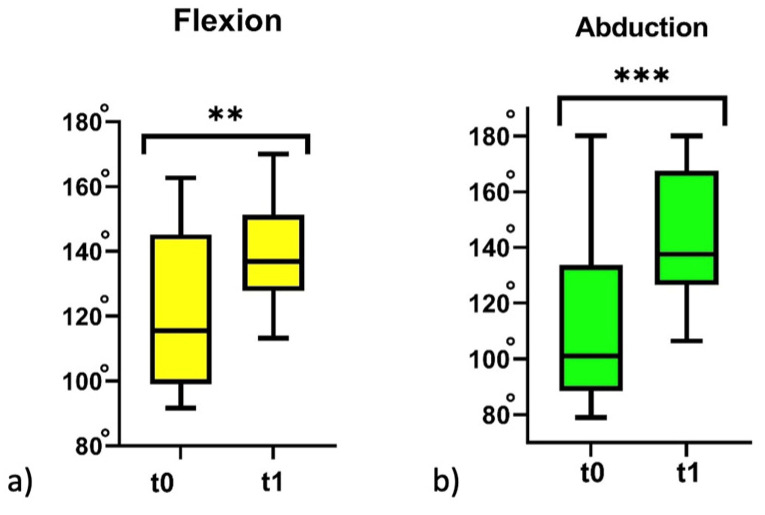
The range of motion of flexion (**a**) and abduction (**b**) in the adhesive capsulitis group before physiotherapy treatment (t0) and after physiotherapy treatment (t1). ** *p* < 0.01; and *** *p* < 0.001; Wilcoxon non-parametric test t0 vs. t1.

Table 1 provides an overview of the main value of RoM in flexion, abduction, and time of activation of the scapula and humerus (Table 1). At baseline (t1), the main value of flexion and abduction in patients with adhesive capsulitis was statistically lower than healthy controls. After 20 sessions of physiotherapy, the adhesive capsulitis group significantly improved the flexion (*p* = 0.001; IC_95%_ −27.8 to −8.0) and abduction of the shoulder (*p* < 0.001; IC _95%_ −48.8 to −18.2) (Figure 1). As such, only flexion remained significantly different from the healthy controls (*p* = 0.001). With regard to the time of activation of the scapula and humerus, it decreased in the scapula (*p* = 0.01; IC_95%_ 0.1 to 1.1), whereas, in the humerus, it did not significantly change (*p* = 0.50; IC_95%_ −0.4 to 0.8). Nevertheless, at the end of the physiotherapy protocol (t1), statistical differences were found between the adhesive capsulitis group and the healthy controls in both the scapula (*p* < 0.01) and humerus (*p* = 0.01).

### 3.2. Pressure Pain Threshold (PPT)

**Figure 2:**
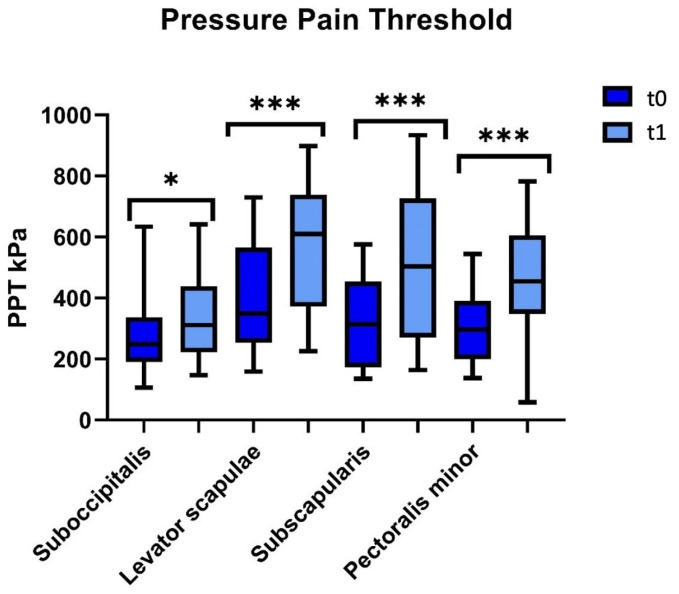
Pressure pain threshold over suboccipitalis, levator scapulae, subscapularis, and pectoralis minor in the adhesive capsulitis group before physiotherapy treatment (t0) and after physiotherapy treatment (t1). * *p* < 0.05 and *** *p* < 0.001; Wilcoxon non-parametric test t0 vs. t1.

Table 2 shows that the main values of the PPT increased statistically significantly in all muscles after 20 sessions of physiotherapy: the sub-occipitalis (*p* = 0.03; IC_95%_ −151.2 to 0.09); levator scapulae (*p* < 0.01; IC_95%_ −268.9 to −83.1); subscapularis (*p* = 0.02; IC_95%_ 275.5 to 111.9); and pectoralis minor (*p* < 0.001; IC_95%_ −217.2 to −73.7). In addition, no differences were found at the end of the physiotherapy treatment (t1) between the adhesive capsulitis group and the healthy controls in the PPT of the sub-occipitalis (*p* = 0.60), levator scapulae (*p* = 0.60), subscapularis (*p* = 0.70), and pectoralis minor (*p* = 0.30) (Table 2) (Figure 2).

## 4. Discussion

Previous studies have highlighted the efficacy of physiotherapy in the decrease in pain and in the improvement in RoM and the functional status of the patients affected by adhesive capsulitis [3,13,38]. In contrast, no previous studies have used digital algometer and inertial sensors together to evaluate the efficacy of the physiotherapy treatment in people with adhesive capsulitis as regards the PPT and RoM. The present study, for the first time, investigated the effect of a specific combined protocol of physiotherapy concerning the quality of movement and pain threshold in patients with adhesive capsulitis with the use of a digital algometer and inertial sensors.

The findings from this study confirm the application of inertial sensors to perform a feasible evaluation and acquisition of the data regarding the kinematics and the RoM of shoulders with adhesive capsulitis.

The first finding of the present study was that the range of motion in flexion and abduction of the shoulder increased significantly after 20 sessions of the combined protocol of physiotherapy. In addition, a more correct timing of activation between the scapula and humerus was restored. Finally, the pressure pain threshold (PPT) increased significantly in all the muscles assessed, and non-statistical differences were found in the PPT with respect to healthy controls after the physiotherapy combined protocol.

With regard to evaluated RoM, this study suggests that physiotherapy can increase the RoM of the shoulder joint in adhesive capsulitis. In fact, after 20 sessions of a combined protocol of physiotherapy, the RoM in flexion and abduction increased significantly. Studies suggest that the loss of mobility of the glenohumeral joint in all directions is related to scapular dyskinesia [42,43,44] and to the pattern of capsular restriction. The improvement in the mobility of the glenohumeral joint is related to the use of manual therapy over the accessory shoulder joint and to the use of proprioceptive neuromuscular facilitation (PNF) techniques with post-isometric relaxation [41]. First, the accessory micro-movements of shoulder joints, such as the acromioclavicular, sternoclavicular, and scapulothoracic joints, are needed prior to performing the macro-movements of the glenohumeral joint [43,45,46]. For this reason, the micro-mobilization of these accessory joints plays a pivotal role in restoring the correct biomechanics of movements. In particular, adhesive capsulitis is associated with scapular dyskinesia [42,43,44,47]. During the shoulder movement, the scapula is elevated prior to the upward rotation due to an incorrect time of activation between the scapula and humerus and due to fascial adhesions between the scapula and thorax. The manual therapy of the scapula could change the tissue pathology of fascial adhesions between the scapula and thorax and restore the upward rotation of the scapula [48]. Second, PNF techniques can stimulate the Golgi tendon organs through autogenic inhibition or post-isometric relaxation, which in turn enhances mobility. In particular, the pattern of capsular restriction is mostly in external rotation, which in turn leads to limitation in flexion and abduction of the shoulder. The isometric contraction in internal rotation followed by relaxation may improve external rotation through the autogenic inhibition of internal rotators and subscapularis muscles. Consequently, the facilitation of external rotation, in turn, improved the range of motion in abduction and flexion [41].

Concerning the time of activation between the scapula and humerus, our study suggests that physiotherapy could reduce scapular dyskinesia and restore the correct scapulohumeral rhythm. In fact, after only 20 sessions of combined protocol of physiotherapy, patients with adhesive capsulitis significantly reduced their time of scapula activation. This result suggests that, after the physiotherapy treatment, the scapula was not elevated prior to the upward rotation due to manual therapy of the scapula followed by active exercise. In fact, after the reduction in fascial adhesions between the scapula and thorax with manual therapy, we used a proprioceptive active exercise to increase the awareness of scapular movement. It seems that this proprioceptive exercise could act both peripherally and centrally [42,49]: peripherally proprioceptive exercise results in a morphological change in the muscle spindle due to metabolic change in the intrafusal muscle fibers; centrally proprioceptive exercise results in a plastic change in the cortex due to correct signals from the mechanoreceptors [42,49]. The association between manual therapy for the fascial adhesion between the scapula and thorax and active proprioceptive exercise for the correct scapular kinematics could restore the correct scapulohumeral rhythm.

On the question of the pressure pain threshold (PPT), we found that the PPT significantly increased in all muscles assessed, and surprisingly, no differences were found at the end of the physiotherapy treatment between the adhesive capsulitis group and healthy controls. A possible explanation for this might be related to the use of manual therapy over the areas biomechanically connected to the shoulder, such as the cervical and dorsal spine. In particular, we used rhythmic oscillatory central posterior–anterior mobilizations on the spinous processes from C2 to D12 segments. It seems that manual therapy, in particular these rhythmic oscillatory mobilizations [40], may have peripheral and central analgesic effects: peripherally, the change in tissue pathology is related to bottom-up mechanisms, such as the stimulation of the peripheral mechanoreceptors and to the inhibition of the nociceptive receptors [28,40,50]; centrally, the change in functional connectivity of the brain area is related to the top-down mechanism, such as pain modulation and body perception [51,52]. Our study suggests that patients with adhesive capsulitis improve more in pain than in range of motion after 20 sessions of the physiotherapy protocol. In fact, although at the end of the physiotherapy treatment, both RoM and PPT improved significantly, only the value of the pressure pain threshold was similar to the healthy controls.

With regard to the limitations of the present work, the most relevant is the absence of an adhesive capsulitis group that did not undergo the proposed physiotherapy protocol. Therefore, it is not possible to completely exclude that some of the observed changes might depend on the time course of the study, being therefore independent of the treatment. In addition, the absence of a long-term follow-up and sex stratification should also be considered. First, a long-term follow-up could highlight the improvement resulting from the physiotherapy treatment over time. Indeed, studies show that continuous treatment over one year could lead to better outcomes and prolonged efficacy in adhesive capsulitis. Second, the sample size did not allow for highlighting sex differences that may be a variable. Furthermore, the small sample size represents a further limitation of the present work. Despite that, our study presents three strong points: firstly, it analyses, for the first time, the effects of a specific combined physiotherapy treatment concerning pain and range of movement outcomes; secondly, it uses a specific protocol ISEO to assess the effect of a specific integrated physiotherapy treatment on the range of motion and scapulohumeral rhythm; thirdly, it compare the main value of pain and range of motion in patients with adhesive capsulitis, before and after treatments, to the main value of healthy controls.

## 5. Conclusions

The use of inertial sensors and a digital algometer seems to be useful for recording the results of a combined physiotherapy treatment in individuals with adhesive capsulitis. The data recorded with these tools suggest that the physiotherapy treatment could be useful both in the decrease in pain and in the improvement in the range of motion. In particular, the association of manual therapy for the fascial adhesion between the scapula and thorax with active proprioceptive exercise for the correct scapular kinematics could restore the correct scapulohumeral rhythm. A randomized control trial with a long-term follow-up could support our findings and highlight the efficacy of prolonged treatment over time. Furthermore, comparing the proposed treatment to a control group of people with adhesive capsulitis who did not participate in the physiotherapy protocol could be helpful in verifying the efficacy of the treatment itself.

## Figures and Tables

**Table 1:** Range of motion with ISEO Motion analysis protocol in adhesive capsulitis group (AD) and healthy controls (HCs).

Motion Analysis Protocol ISEO	AD t0	AD t1	HCs
Flexion RoM (degrees)	120.9 ± 23.1 ^###^	138.8 ± 16 ** ^###^	155.4 ± 12.2
Abduction RoM (degrees)	111.7 ± 28.4 ^###^	145.2 ± 23.3 ***	158.2 ± 11
Time activation Scapula (s)	2.9 ± 1.1 ^###^	2.3 ± 0.9 * ^##^	1.6 ± 0.6
Time activation Humerus (s)	2.8 ± 1.2 ^##^	2.6 ± 1 ^##^	1.7 ± 0.7

* *p* < 0.05; ** *p* < 0.01; and *** *p* < 0.001; Wilcoxon non-parametric test t0 vs. t1 in adhesive capsulitis group (AD). ^##^ *p* < 0.01; and ^###^ *p* < 0.001; Mann–Whitney U test comparing AD versus healthy controls (HCs).

**Table 2:** Pressure pain threshold (PPT) in adhesive capsulitis group (AD) and healthy controls (HCs).

Pressure Pain Threshold	AD t0	AD t1	HCs
Sub-occipitalis (kPa)	262.5 ± 125.2 ^#^	338.1 ± 131.7 *	387.5 ± 187.8
Levator Scapulae (kPa)	397.5 ± 180.2 ^##^	573.6 ± 212.6 ***	621.2 ± 223.2
Subscapularis (kPa)	323 ± 144.5 ^#^	516.8 ± 248.1 ***	482.4 ± 210.4
Pectoralis Minor (kPa)	313.5 ± 114 ^###^	459 ± 186.4 ***	549.3 ± 205.7

* *p* < 0.05 and *** *p* < 0.001; Wilcoxon non-parametric test t0 vs. t1 in adhesive capsulitis group (AD). ^#^ *p* < 0.05; ^##^ *p* < 0.01; and ^###^ *p* < 0.001; Mann–Whitney U test comparing AD versus healthy controls (HC).

## Data Availability

Anonymized data can be obtained upon reasonable request by contacting the corresponding author.

## Supplementary Materials

The following supporting information can be downloaded at https://www.mdpi.com/article/10.3390/jfmk8040142/s1.
