# Supplementary material for: Inertial Sensors and Pressure Pain Threshold to Evaluate People with Primary Adhesive Capsulitis: Comparison with Healthy Controls and Effects of a Physiotherapy Protocol
Source: J Funct Morphol Kinesiol. 2023 Oct 6;8(4):142. doi: 10.3390/jfmk8040142 (PMC10594492; doi:10.3390/jfmk8040142)
Supplement: Supplementary file 1 [file jfmk-08-00142-s001.zip › jfmk-2609630-supplementary.docx]

**ISEO protocol**

The ISEO protocol (INAIL shoulder and elbow outpatient protocol) is a movement analysis protocol that uses magnetic-inertial sensors to measure the kinematics of the upper limb. ISEO is a published peer-reviewed protocol available for scapulohumeral rhythm detection with IMMS technology. To detect this data, the protocol involves the use of an Xbus kit (Xsens Technologies NL), composed of 4 MTw sensors.

For the application of the ISEO protocol the following steps must be respected:

1. Place the MTw sensors on the predefined landmarks:

- chest: the MTw sensor is positioned at the level of the manubrium of the sternum;
- scapula: the MTw sensor is positioned immediately above the spine of the scapula in its middle third between the acromial process and the trigonum spinae (it is important that it is aligned with the spine)
- humerus: the MTw sensor is positioned above the middle third of the slightly posterior arm;
- forearm: the MTw sensor is positioned in the distal third of the forearm.

1. Define the anatomical reference system. In this regard, the calibration of the sensors is carried out in a static position that the subject must maintain: standing position, elbow flexed at 90°, thumb pointing upwards to ensure neutral rotation of the forearm, humerus along the side perpendicular to the floor in neutral position.
2. Definition of the functional axes. Elbow flexion-extension and pronation-supination movements are performed in order to define the axes on which these movements occur.
3. Calculation of the kinematics of the joint. The orientation of the scapula and humerus are expressed relative to the thorax. The orientation of the scapula is defined in terms of protraction-retraction (PR-RE), medio-lateral rotation (ME-LA) and anterior-posterior tilt (P-A). The orientation of the humerus is defined in terms of flexion-extension (FL-EX), abduction-adduction (AB-AD) and internal and external rotation (IN-EX).
